# Fully automated diaphragm segmentation on routine 2D-cine and 3D thoracic MRI: multireader validation

**DOI:** 10.1186/s41747-026-00805-z

**Published:** 2026-10-16

**Authors:** Samia Boussouar, Alexiane Pasquier, Damien Vaurs, Arthur Dilane Douho Kamta, Anna Reitmann, Nicoletta Pasi, Victoria Donciu, Sara Benchara, Tatiana Wong, Myriam Belghiti, Ithar Gharmaoui, Rose-Marie Dubuisson, Angéline Nemeth, Vincent Lebon, Claire Pellot-Barakat, Marie Poirier-Quinot, Xavier Maître, Alban Redheuil, Dima Rodriguez

**Affiliations:** 1 Department of Cardiothoracic Imaging, Pitié-Salpêtrière University Hospital AP-HP, Sorbonne University, Paris, France; 2 Paris-Saclay University, CEA, CNRS, Inserm, BioMaps, Orsay, France; 3 ICAN Foundation for Innovation in Cardiometabolism and Nutrition, Paris, France; 4 Specialized and Emergency Imaging Department, Pitié-Salpêtrière University Hospital AP-HP, Sorbonne University, Paris, France; 5 Sorbonne University, INSERM 1146, CNRS 7371, Laboratoire d’Imagerie Biomédicale, Paris, France

**Keywords:** Automatic segmentation, Deep learning, Diaphragm, Magnetic resonance imaging, Thoracic imaging

## Abstract

**Objective:**

The diaphragm is central to respiratory mechanics and may be impaired in various diseases. Parametric magnetic resonance imaging (MRI) assessment is limited by time-consuming manual segmentation. We developed and validated fully automated diaphragm segmentation on two-dimensional (2D)-cine and three-dimensional (3D) thoracic MRI.

**Materials and methods:**

In this single-center study, eligible adults without known diaphragmatic disorders underwent 2D-cine MRI, 3D MRI, or both. Two U-Net models (2D/3D) were trained using expert manual contours as ground truth. Performance was evaluated with normalized surface Dice (NSD) with 3-voxel tolerance and average surface distance (ASSD). In the main cohort, five blinded radiologists rated segmentation usability and image quality. An additional separate pathology cohort was assessed by two blinded readers using the same scale.

**Results:**

Overall, 59 individuals contributed 70 MRI acquisitions: 29 women, 30 men; aged 49.3 ± 18.8 years (mean ± standard deviation). Inference required 5 s (2D) and 30 s (3D) *versus* 10 min and 4.5 h for manual annotation. Segmentation accuracy was high: NSD 0.95 ± 0.08 (2D) and 0.92 ± 0.06 (3D), with ASSD 1.97 ± 2.29 mm (2D) and 1.88 ± 1.97 mm (3D). In the main cohort, segmentations were rated clinically usable in 87.5% (2D) and 100% (3D). Mean image-quality scores were 4.0/5 (2D) and 4.2/5 (3D) and correlated with segmentation quality. In the pathology cohort (*n* = 5), segmentation usability for 2D images was 75% for reader 1 and 70% for reader 2 for 2D images and 80% for both readers for 3D volumes.

**Conclusion:**

Automated diaphragm segmentation on 2D-cine and 3D MRI is feasible, accurate, and time-efficient, supporting scalable, standardized diaphragm analysis as a basis for deriving diaphragm biomarkers.

**Key Points:**

***Question*** Can fully automated diaphragm segmentation make routine 2D-cine and 3D thoracic MRI analysis clinically feasible by replacing time-consuming manual delineation?

***Findings*** Automated segmentation achieved normalized surface Dice above 0.9, radiologist acceptability in 87.5–100% of cases, and inference times below 30 s

***Relevance statement*** Automated diaphragm segmentation on routine 2D-cine and 3D static MRI yields accurate, radiologist-acceptable segmentations with normalized surface Dice exceeding 0.9 and mean surface deviations below 2 mm. This approach may support standardized diaphragm assessment for objective quantification in thoracic and neuromuscular imaging pending larger multicenter validation.

**Graphical Abstract:**

**Figure:**
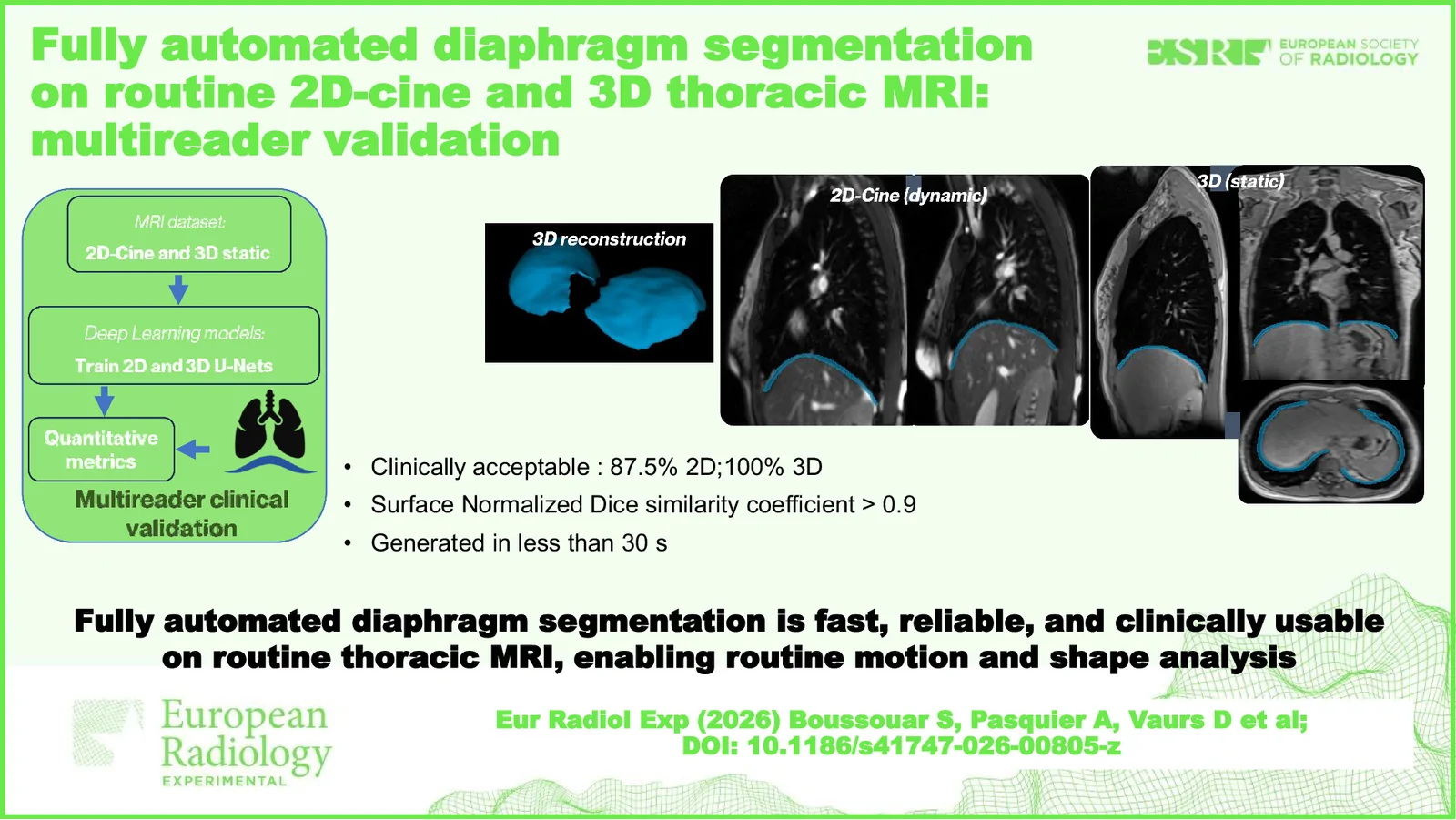

## Background

The diaphragm is the principal respiratory muscle and a central determinant of thoracoabdominal mechanics. Dysfunction arises in a wide range of conditions, including neuromuscular disorders, chronic respiratory diseases, oncologic treatment-related toxicities, inflammatory myopathies, and abdominal disorders that alter diaphragmatic loading [1]. Conventional diagnostic tools remain limited: fluoroscopy and CT provide direct visualization but involve ionizing radiation exposure and predominantly static or limited-angle assessments.

Ultrasound is radiation-free but highly operator-dependent and often limited to two-dimensional (2D) measurements [2]. Magnetic resonance imaging (MRI) offers a noninvasive, radiation-free alternative with excellent soft-tissue contrast, enabling detailed 2D-cine and three-dimensional (3D)/four-dimensional (4D) analysis of diaphragmatic morphology and motion [3, 4]. Recent applications in rare diseases such as thoracic insufficiency syndrome and Pompe disease have demonstrated that MRI-derived diaphragm metrics can reveal subtle functional decline not detectable by conventional spirometry, underscoring the sensitivity and potential clinical relevance of this approach [5, 6].

Despite these advances, diaphragmatic MRI analysis still predominantly relies on manual or semiautomated segmentation and cumbersome phase selection, which limits surface modeling, reproducibility, scalability, and clinical translation [7]. Fully automated volumetric diaphragm segmentation remains a key unmet need for dynamic diaphragm MRI. If robust, it could enable standardized, reader-independent assessment of diaphragm morphology and motion from routine thoracic MRI, reduce manual workload, and improve measurement reproducibility within existing clinical workflows.

The aim of the present study is to develop and validate a fully automated framework for diaphragm segmentation in MRI.

## Materials and methods

### Study design and population

This single-center study was approved by the local institutional review board (“gb-Spiro 3D” CPP 22.00822.000074), and written informed consent was obtained from all participants. Data were de-identified, and the study adhered to the Declaration of Helsinki and Good Clinical Practice.

The study comprised retrospective and prospective components. Inclusion criteria were adults undergoing clinically indicated thoracic MRI with available 2D-cine MRI, 3D volumetric interpolated breath-hold examination (VIBE) MRI, or both. Exclusion criteria included known or suspected diaphragmatic dysfunction on medical record review and nondiagnostic image quality. Image quality and exclusion criteria were assessed by an experienced thoracic radiologist (> 10 years of experience) using predefined criteria based on diagnostic usability and the presence of severe artifacts.

For the retrospective component, consecutive clinical 2D-cine MRI acquisitions performed between September 2023 and September 2025 were screened. For the prospective component, after implementation of the 3D VIBE T1-weighted gradient-echo (T1W-GRE) MRI sequence in July 2025, consecutive individuals undergoing MRI between July 2025 and October 2025 were prospectively screened.

Study eligibility was defined at the individual level, whereas model development was defined at the MRI acquisition level. The 2D-cine dataset included all eligible 2D-cine MRI acquisitions, and the 3D dataset included all eligible 3D VIBE MRI acquisitions obtained after implementation of the sequence. Because sequence availability and image quality were assessed separately for each MRI acquisition, an individual could contribute to the 2D-cine dataset only, the 3D dataset only, or both.

### MRI acquisition protocol

MRI acquisitions were performed on a 1.5-T Magnetom Sola MRI (Siemens Healthineers, Erlangen, Germany) using a 2D-cine sequence and a 3D VIBE T1w-GRE sequence, with the field of view individually adapted to cover the thoracic cage.

For 2D MRI acquisitions, a cine balanced steady-state free precession (bSSFP) multislice sequence was acquired (native voxel size: 1.6 × 1.6 with 6-mm slice thickness). Based on coronal localizers, three sagittal slices were positioned to capture each hemidiaphragm and thoracic motion. Subjects performed forced inspiration and expiration throughout the 100-s acquisition.

For 3D MRI acquisitions, the VIBE T1w-GRE sequence was acquired in the sagittal plane, with a native voxel size of 1.4 × 1.4 × 2 mm. The acquisition was performed twice, with 14-s breath-holds at end-inspiration and end-expiration. Typical acquisition parameters are summarized in Supplemental Table S1.

### Reference standard

Manual annotations were performed in 3DSlicer (version 5.8) by a thoracic radiologist with 13 years’ experience. Diaphragmatic pillars were excluded due to incomplete coverage. For the 2D dataset, annotations were performed manually slice-by-slice (Supplemental Fig. S2). For the 3D dataset, the output of a pre-segmentation model trained on a separate dataset (not as an endpoint and acquired on an external cohort; see Supplemental Material S3) served as a visual guide for the radiologist, contouring was initiated on the native sagittal view and systematically refined using multiplanar (axial and coronal) views and final reference masks were produced after full manual refinement (Supplemental Figs. S3 and S4).

### Data preprocessing and dataset splitting

MRI intensity values were standardized independently for each volume using z-score normalization (zero mean, unit variance) based on that volume’s mean and standard deviation before processing. For 3D model input, 3D volumes were resampled from their native voxel spacing (Supplemental Table S1) to 1.4-mm isotropic voxels using trilinear interpolation. Corresponding manual segmentation masks were resampled using nearest-neighbor interpolation.

**Fig. 1:**
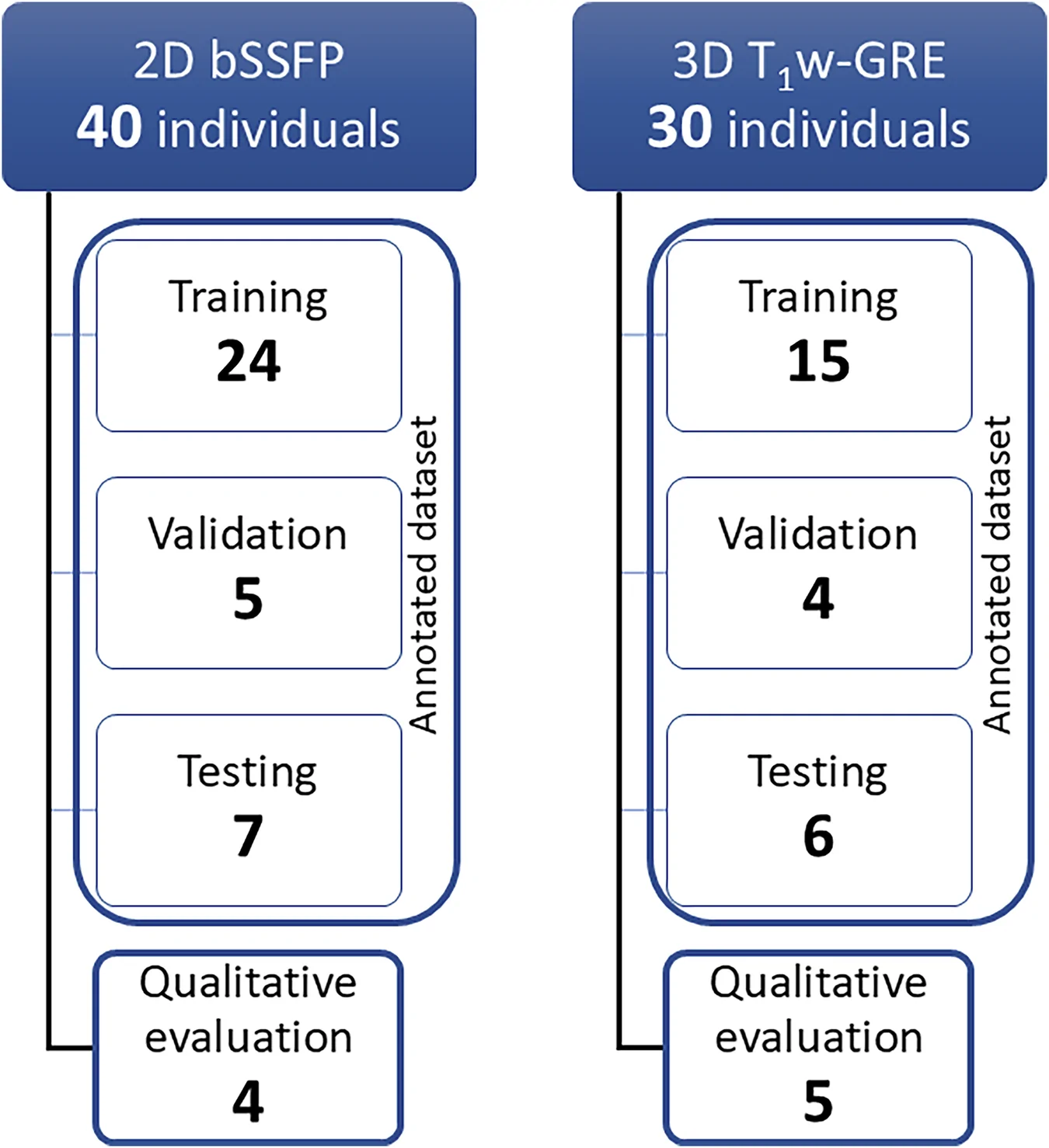
Overview of the dataset split. Distribution of the 70 MRI acquisitions across training/validation/testing (annotated) and the independent qualitative evaluation set

To address graphics processing unit memory constraints, patch-based training used 48 × 48 voxels (2D) and 48 × 48 × 48 voxels (3D). Dataset creation and division into training, validation, and test sets are described in Supplemental Table 2 and Supplemental Figs. S5–S8. All splits were individual-disjoint: no individual contributed to more than one subset. The distribution of the data across training, validation, and testing sets is illustrated in Fig. 1.

### U-Net model training and validation strategy

Two models with the same U-Net design [8] were developed (Supplemental Material S3). Optimization used a hybrid loss function combining Dice and binary focal cross-entropy, the latter enhancing sensitivity to sparse positive voxels. Model training was monitored using validation loss to prevent overfitting, and performance was assessed with five-fold participant-level cross-validation. Each model was trained and tuned independently, then evaluated on separate held-out test sets, with no sharing of images or labels.

### Testing and evaluation metrics

Final model performance was evaluated on an independent held-out test set. For the 2D model, a crop of 20 lines at the top and bottom of the images was applied before inference to restrict the field of view. Inference was performed on each model using a patch size of 48 × 48 (48 × 48 × 48 for 3D) and a stride of 8 × 8 (8 × 8 × 8 for 3D). Prediction probabilities were thresholded using validation-tuned values (0.4 for 2D, 0.5 for 3D). Primary endpoints were ASSD and NSD at tolerances of 1, 3, and 5 voxels, complemented by HD95. Metrics were computed on extracted surfaces in physical units and summarized as mean ± standard deviation (SD).

### Qualitative evaluation

Five board-certified radiologists (> 10 years of experience, *n* = 2; 5–10 years, *n* = 2; < 5 years, *n* = 1) independently evaluated segmentations after calibration, using data outside training/validation/testing (Fig. 1). Neither of them was involved in the study design and model development. In 2D-cine MRI, each hemidiaphragm was evaluated at two respiratory phases (end-inspiration and end-expiration); in 3D VIBE MRI, both hemidiaphragms were assessed jointly at both phases. Two grids were used: (1) SegQ (1 = not usable, 2 = major revision required, 3 = minor revision required, 4 = acceptable as it is) and (2) a five-item binary checklist (anatomical adherence, continuity, interfaces, major errors, temporal consistency). Image quality (IQ) was rated on a 5-point IQ Likert scale (from 1 = nondiagnostic to 5 = excellent) adapted from RI-QUAL [9]. The evaluation framework is summarized in Table 1 and Supplemental Table S4. Clinical acceptability required SegQ ≥ 3 and checklist ≥ 3/5, by majority consensus (≥ 3/5 readers). Only raw images, model segmentations, and the reading grid were provided. Scores were summarized across readers for each evaluated condition (2D: hemidiaphragm × phase; 3D: volume × phase).

**Table 1:** Qualitative and image-quality assessment tools

Tool	Scale/items	Definition
Global 4-point scale (SegQ)	1 = not usable 2 = major revision required 3 = minor revision required 4 = acceptable as is	Overall quality of segmentation
Five-item checklist	(1) Anatomical adherence (2) 2D/3D continuity (3) Anatomical interface (4) Absence of major errors (holes, leakage) (5) Temporal consistency	Binary scoring (Yes/No) for each criterion (1) Segmentation respects adjacent structures. Correct anatomical interfaces (liver, spleen, chest wall) (2) Segmentation remains continuous across slices and planes (3) Boundary follows the true diaphragmatic contour (4) No holes, discontinuities, or leakage into adjacent regions (5) Stable segmentation without abrupt changes
Image quality (IQ)	5-point Likert scale (1 = nondiagnostic/5 = excellent)	5 = Excellent: No artifacts, full confidence, diagnostic. 4 = Good: Mild artifacts, unlikely impact (< 10%). 3 = Adequate: Moderate artifacts, possible impact (~ 50%). 2 = Poor: Marked artifacts, probable impact (~ 75%). 1 = Nondiagnostic: Severe artifacts, segmentation unusable.

The table summarizes the three evaluation components used in this study: a global 4-point segmentation-quality scale, a five-item checklist for anatomical and temporal consistency, and an image-quality (IQ) score adapted from the RI-QUAL framework [9]. A color-coded version of the IQ scoring card is provided in Supplemental Table 4 for visual reference

### External pathology feasibility cohort

Segmentation was evaluated in five patients with diaphragmatic dysfunction as a feasibility assessment using the same 2D-cine and 3D protocols. These cases were not used for training, validation, or model evaluation.

### Qualitative assessment (pathology feasibility cohort)

Two thoracic radiologists (> 10 years of experience) independently rated image and segmentation quality using the same procedure as in the main cohort. One reader was not involved in reference labeling/model development and had not previously reviewed the cases.

## Results

First, we describe the study cohort. We then report quantitative performance metrics and finally the qualitative radiologist evaluation.

### Study cohort

A total of 246 consecutive clinical 2D-cine MRI acquisitions were screened retrospectively. After exclusion of individuals with known or suspected diaphragmatic dysfunction and acquisitions with nondiagnostic image quality, 40 analyzable 2D-cine MRI acquisitions were retained.

Following the implementation of the 3D VIBE T1w-GRE sequence, 35 consecutive 3D VIBE acquisitions were obtained prospectively. Of these, 5 were excluded because image quality was considered nondiagnostic for reliable visual assessment, leaving 30 analyzable 3D VIBE MRI acquisitions.

Therefore, the final imaging consisted of 40 2D-cine MRI acquisitions and 30 3D VIBE MRI acquisitions. Overall, 59 individuals contributed to at least one imaging dataset: 29 contributed to the 2D-cine dataset only, 19 to the 3D VIBE dataset only, and 11 to both datasets (Supplemental Fig. S1). In the overall cohort (*n* = 59), 29 individuals were women and 30 were men. Mean age was 49.3 ± 18.8 years, mean height was 168.6 ± 8.3 cm, and mean weight was 71.1 ± 14.0 kg. Among individuals with 2D cine MRI (*n* = 40), 21 were women and 19 were men. Mean age was 47.7 ± 17.5 years, mean height was 167.8 ± 8.0 cm, and mean weight was 70.4 ± 14.9 kg. Among individuals with 3D VIBE MRI (*n* = 30), 14 were women and 16 were men. Mean age was 45.2 ± 21.1 years, mean height was 169.1 ± 8.9 cm, and mean weight was 70.1 ± 12.2 kg. For the external pathology feasibility cohort, 5 patients were included with both 2D-cine and 3D VIBE MRI. The mean ± SD age was 53.4 ± 18.6 years, with 4 men and 1 woman. The mean ± SD weight and height were 82.3 ± 5.0 kg and 169.5 ± 10.2 cm, respectively.

### Automated segmentation performance

**Fig. 2:**
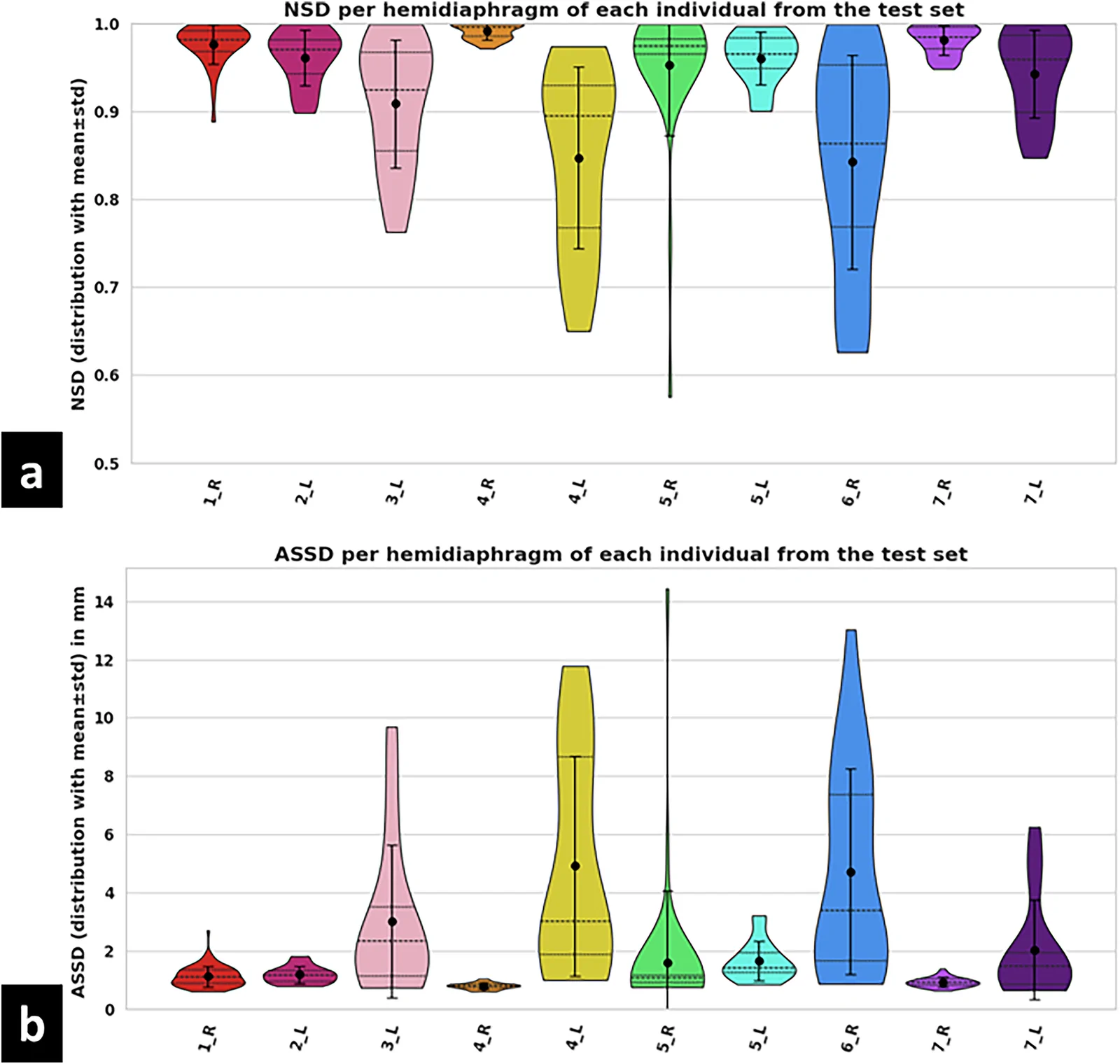
Slice-wise dispersion of performance metrics (2D model test set). Violin plots show the distribution of (**a**) NSD (normalized surface Dice; 3-voxel tolerance; higher is better) and (**b**) ASSD (average surface distance in mm; lower is better) across slices for each hemidiaphragm and individuals. Within each violin, the dot and horizontal bar denote the mean and ± 1 standard deviation, respectively, while the width of the violin illustrates the data distribution density

Quantitative performance was evaluated by comparing the computed output against the manual reference masks on the independent test set using surface-based metrics. NSD is reported at 1-, 3-, and 5-voxel tolerances (NSD-1/3/5), with ASSD (mm) and HD95 (mm). Unless otherwise specified, NSD refers to 3-voxel tolerance. Quantitative test set results are summarized in Table 2. Results for the 2D model are illustrated in Fig. 2.

**Table 2:** Results of the testing set for the surface metrics (NSD) and distance (ASSD and HD95), mean ± SD

Model	NSD-1	NSD-3	NSD-5	ASSD (mm)	HD95 (mm)
2D	0.787 ± 0.093	0.945 ± 0.076	0.961 ± 0.072	1.97 ± 2.29	9.89 ± 15.2
3D	0.835 ± 0.071	0.922 ± 0.057	0.946 ± 0.048	1.88 ± 1.97	7.67 ± 6.27

Cross-validation showed consistent validation performance with low inter-fold variability. For reference, mean ± SD training loss across five folds was 0.173 ± 0.019 for the 3D model and 0.245 ± 0.004 for the 2D model.

**Fig. 3:**
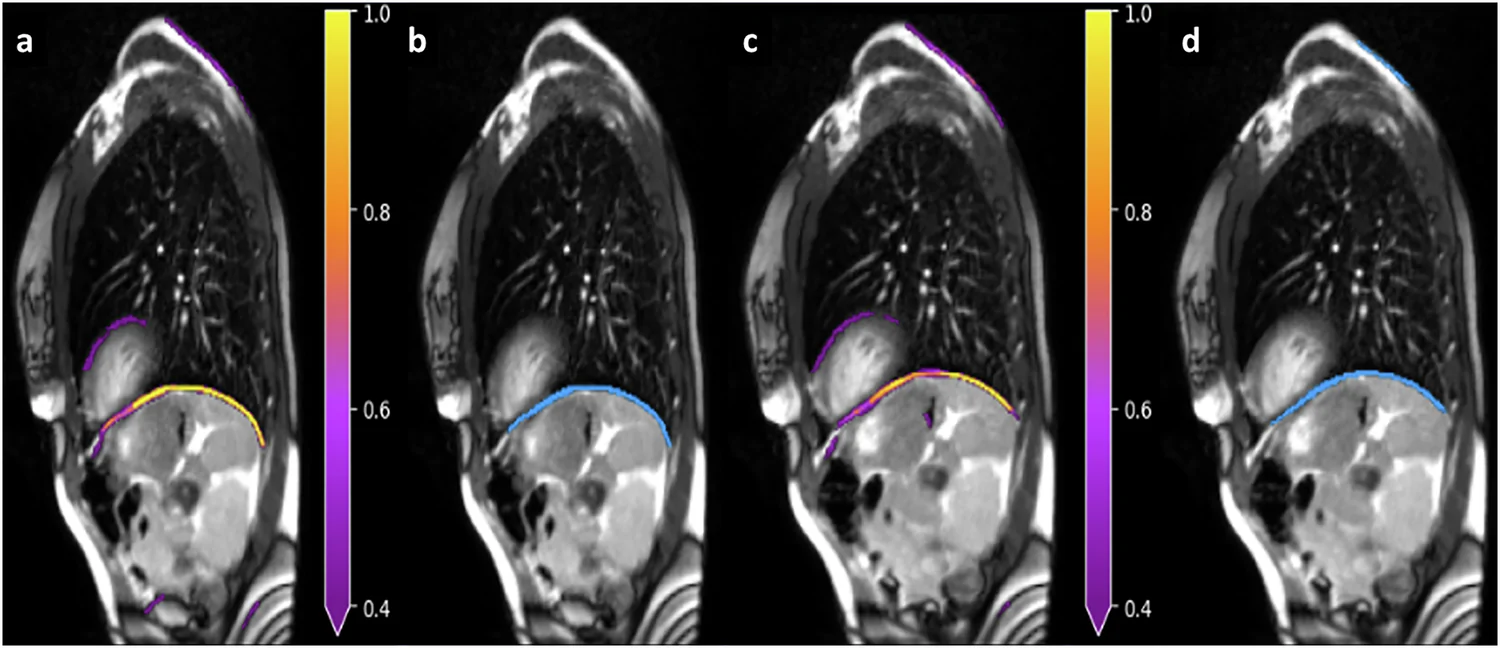
Example 2D model output. Probability maps (**a**, **c**) and corresponding binary masks thresholded at 0.4 (**b**, **d**). **b** shows good agreement (NSD = 0.92), whereas **d** illustrates shoulder misclassification (NSD = 0.66). Color bar indicates probability (0.4–1.0). NSD, Normalized surface Dice

**Fig. 4:**
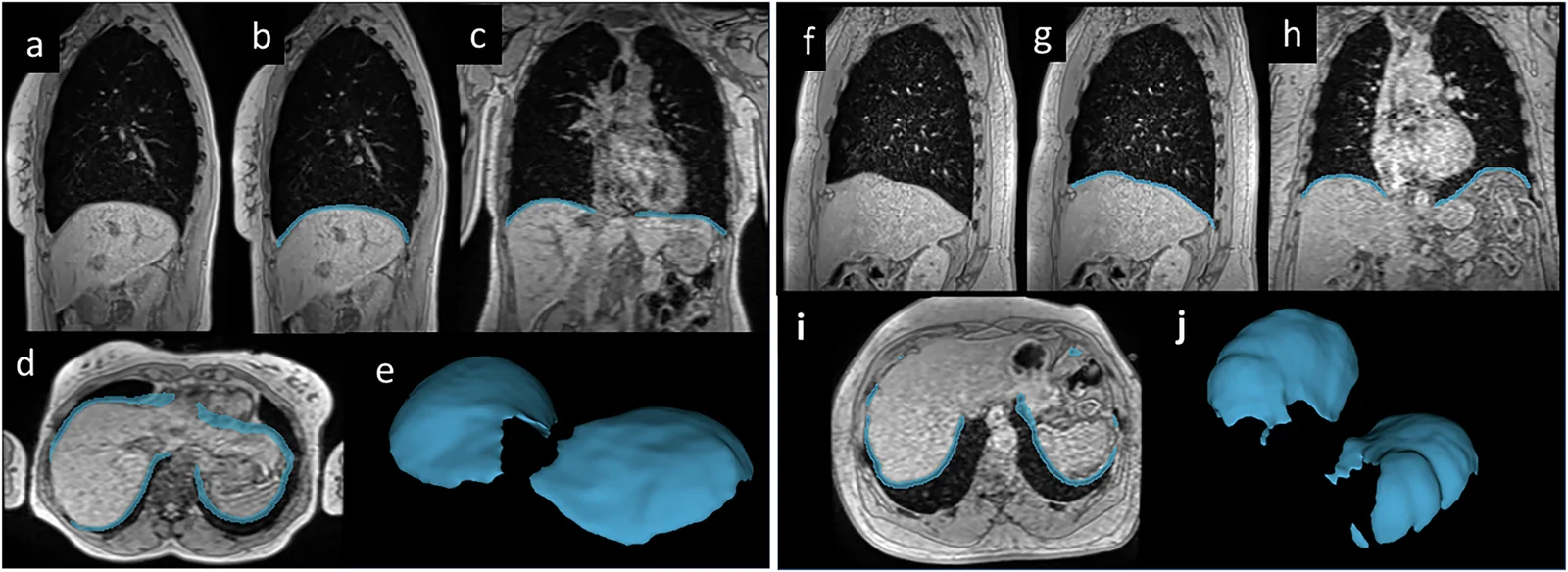
3D model: exemplar of high- *versus* low-performance cases. Left panel (**a**–**e**): individual with NSD = 0.944. **a**–**c** show orthogonal views in which the predicted diaphragm boundary (blue) closely matches the reference across the domes and costophrenic recesses. **d** shows a representative axial slice with tight overlap, and **e** shows the post-threshold 3D surface, demonstrating a smooth, continuous reconstruction of both hemidiaphragms. Right panel (**f**–**j**): individual with NSD = 0.877. **f**–**h** illustrate typical failure modes, including under-segmentation near the anterior/lateral recesses, minor overreach toward the liver–heart interface, and discontinuities across the central tendon. **i** highlights slice-wise misalignment on an axial view, and **j** shows a fragmented, less regular 3D surface. Overall, higher NSD is associated with accurate, smooth, and complete dome delineation (**a**–**e**), whereas lower NSD reflects boundary mismatches and surface irregularities (**f**–**j**), particularly in the presence of motion, through-plane partial-volume effects, and reduced soft-tissue contrast. NSD, Normalized surface Dice

Examples of high and low-performance segmentations are shown (Figs. 3 and 4), and 2D and 3D videos are provided in Supplemental Videos 1 and 2.

### Qualitative radiologist evaluation (main cohort)

Qualitative results are summarized in Table 3 (2D) and in Table 4 (3D) using SegQ and IQ ratings, the checklist-based composite score, and the proportion of clinically acceptable segmentations (as defined in “Methods”).

**Table 3:** Qualitative radiologist evaluation of 2D-cine diaphragm segmentations for four individuals, assessed for both hemidiaphragms at inspiratory and expiratory phases

Individuals Hemidiaphragm Inspiration (I)/expiration (E)	1				2				3				4				Mean ± SD
	Right		Left		Right		Left		Right		Left		Right		Left		
	I	E	I	E	I	E	I	E	I	E	I	E	I	E	I	E	
Radiologist A	8	8	5	5	7	9	5	7	8	8	9	8	9	8	8	8	7.5 ± 1.4
Radiologist B	6	8	2	2	9	9	7	9	9	9	7	9	9	9	7	7	7.4 ± 2.3
Radiologist C	9	7	9	3	9	9	7	7	7	9	9	7	9	8	6	6	7.6 ± 1.7
Radiologist D	7	6	7	4	7	9	6	5	9	9	9	9	9	9	8	6	7.4 ± 1.7
Radiologist E	9	7	2	2	7	9	3	3	7	9	8	7	9	9	6	6	6.4 ± 2.6
Mean	7.8	7.2	5	3.2	7.8	9	5.6	6.2	8	8.8	8.4	8	9	8.6	7	6.6	7.3 ± 1.6
IQ	3.8	3.8	3.6	3.2	4.2	4.2	3.8	3.6	4.8	4.8	4.8	4.6	3.8	3.8	3.8	3.8	4.0 ± 0.5

Cells report the composite qualitative score (SegQ + 5-item checklist; range 1–9) assigned by each reader. The last rows summarize the mean score across readers for each condition and the corresponding image-quality (IQ) score (range 1–5). Clinical acceptability was adjudicated by majority consensus (≥ 3/5 readers); composite scores are shown

**Table 4:** Qualitative radiologist evaluation of 3D diaphragm segmentations for five individuals at inspiratory and expiratory phases

Reader Inspiration (I)/expiration (E)	1E	2I	2E	3I	3E	4I	4E	5I	5E	Mean ± SD
Radiologist A	8	6	5	9	8	9	8	8	8	7.7 ± 1.3
Radiologist B	7	5	5	9	9	8	7	7	9	7.3 ± 1.6
Radiologist C	6	7	6	9	6	6	8	7	7	6.9 ± 1.1
Radiologist D	8	7	9	8	9	8	8	7	7	7.9 ± 0.8
Radiologist E	7	5	5	9	8	6	6	7	9	6.9 ± 1.5
Mean	7.2	6.0	6.0	8.8	8.0	7.4	7.4	7.2	8.0	7.3 ± 0.9
IQ	4.0	3.4	3.8	4.8	5.0	4.8	5.0	4.2	3.2	4.2 ± 0.7

Cells report the composite qualitative score (SegQ + 5-item checklist; range 1–9) assigned by each reader. The last rows summarize the mean score across readers for each case and the corresponding image-quality (IQ) score (range 1–5). Clinical acceptability was adjudicated by majority consensus (≥ 3/5 readers); composite scores are shown

#### 2D model

**Fig. 5:**
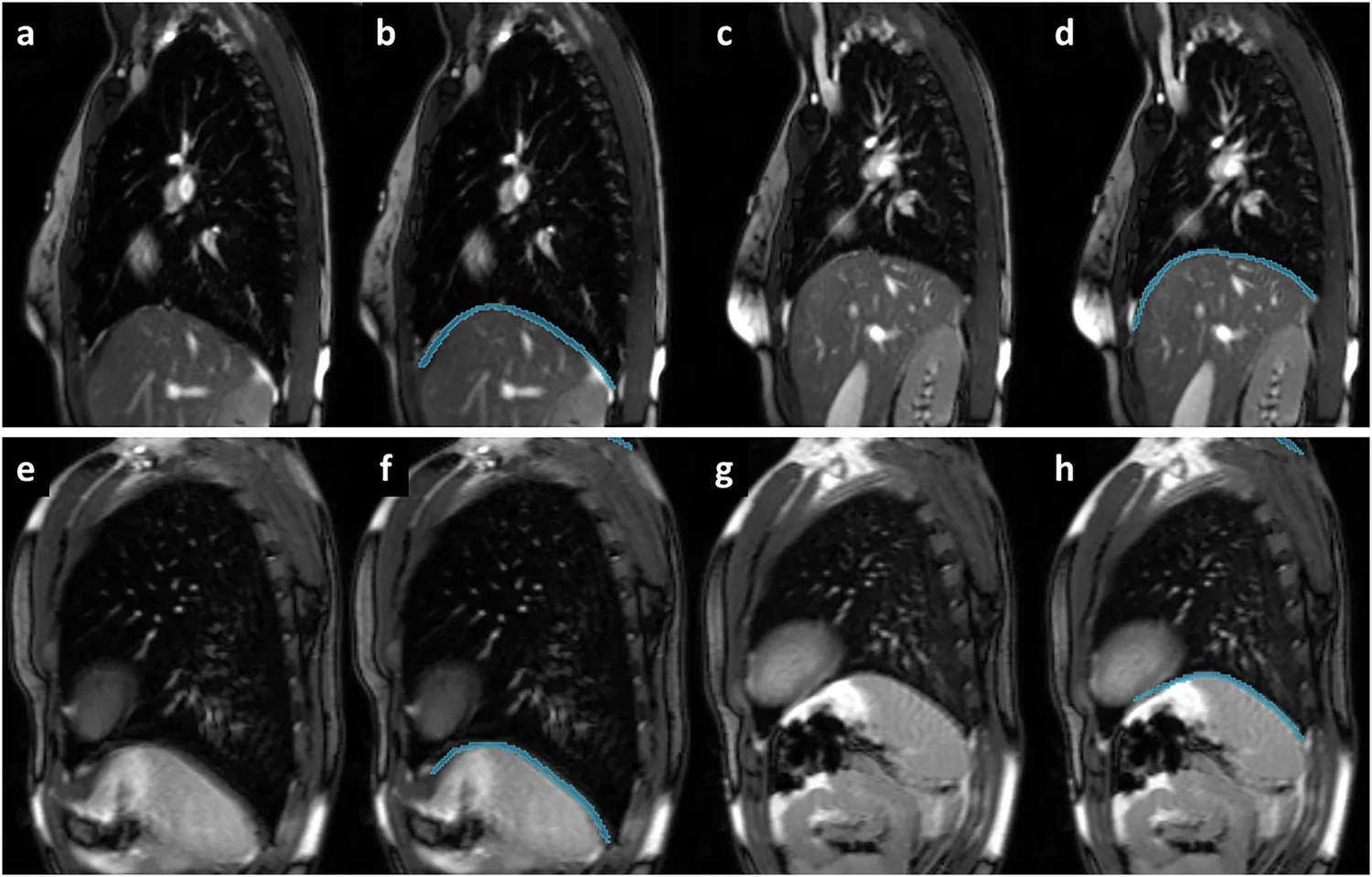
2D model: exemplar of high- *versus* low-performance cases. For one representative individual, the top row shows the right hemidiaphragm and the bottom row the left hemidiaphragm, at end-inspiration (**a**, **b**, **e**, **f**) and end-expiration (**c**, **d**, **g**, **h**). Blue overlays depict the automated diaphragm boundary. Note that the contours do not extend fully to the lateral edges. In **h**, a small region of the shoulder–chest-wall interface, spatially distant from the diaphragm, was incorrectly included. Overall, the predicted boundaries remain continuous and well aligned with the diaphragm–lung interface, without holes or other implausible segmentations

2D images segmented by the model were analyzed (inspiratory and expiratory phases for both right and left hemidiaphragms), yielding 16 segmentations across 4 participants. SegQ/IQ/checklist was applied as defined in “Methods” and in Table 1. The mean ± SD global segmentation (SegQ) score was 3.4 ± 0.7 out of 4, and the mean IQ score was 4.0 ± 0.5 out of 5, yielding a composite qualitative score (SegQ + 5-item checklist, range 1–9) of 7.3 ± 1.6. Qualitative results for 2D segmentations are summarized in Table 3. Clinically acceptable segmentations, defined as global SegQ ≥ 3 and checklist ≥ 3/5, were adjudicated in 87.5% of cases for radiologists (14/16). Representative examples of high- and low-performance 2D segmentations are shown in Fig. 5. Correlation analysis demonstrated a moderate-to-strong positive association between image quality and segmentation performance (*r* = 0.65, *p* = 0.006).

#### 3D model

**Fig. 6:**
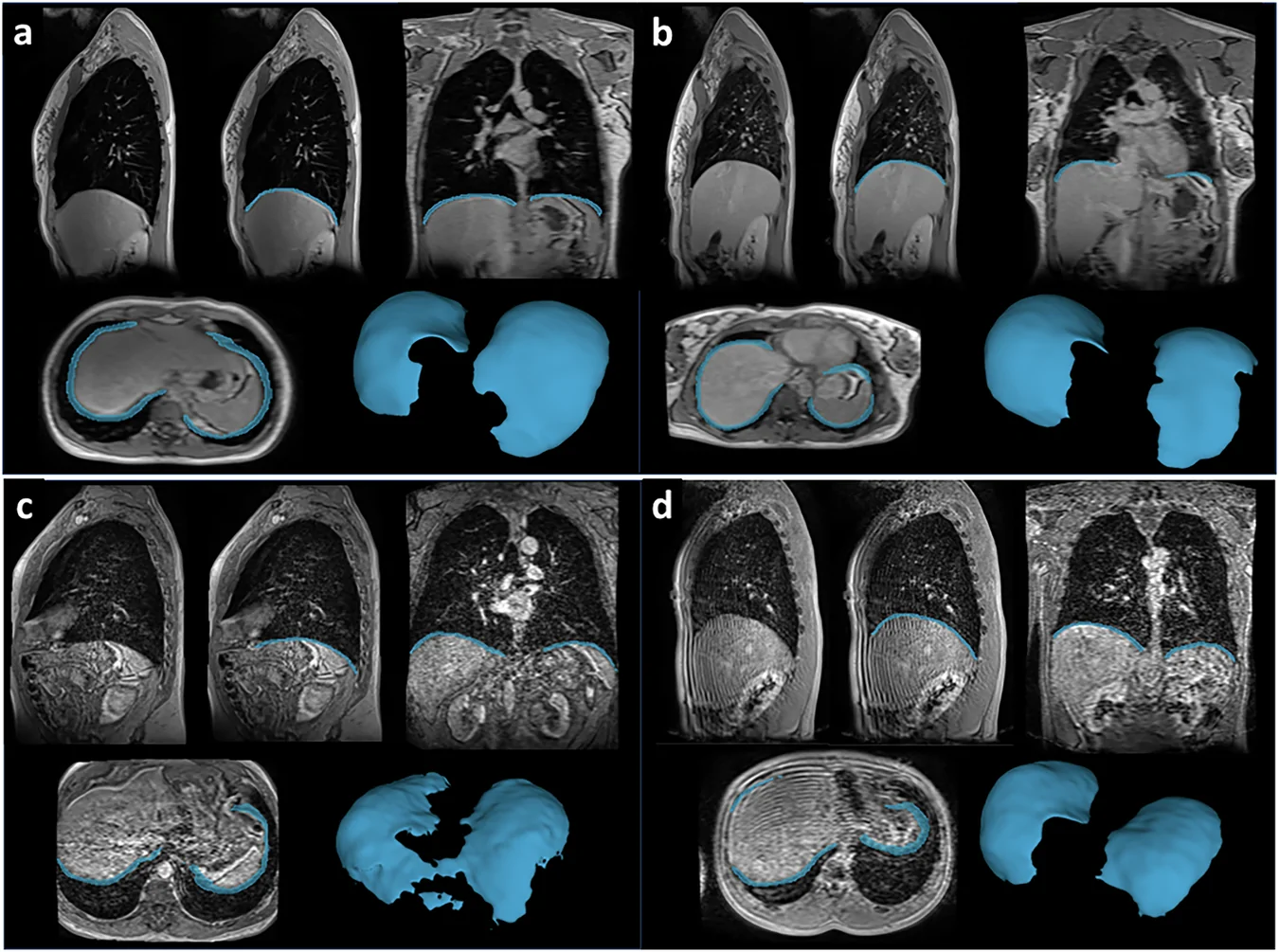
Qualitative reader evaluation for different image qualities (3D model). For each case, orthogonal views with predicted boundary (blue), a representative axial slice, and the 3D surface are shown. **a** Individual 3, end-inspiration (IQ = 5.0; composite score = 8.0). **b** Individual 3, end-expiration (IQ = 3.8; composite score = 6.0). **c** Individual 2, end-inspiration (IQ = 3.2; composite score = 8.0). **d** Individual 5, end-expiration (IQ = 3.6; composite score = 6.2). Higher composite scores correspond to more accurate and continuous dome reconstructions

3D volumes segmented by the model were analyzed (inspiratory and expiratory breath-holds with both hemidiaphragms included in each volume), yielding 9 segmentations across 5 participants. One end-expiratory 3D volume was excluded due to strong motion artifacts; the corresponding end-inspiratory volume was suitable and therefore included in the analysis. The mean ± SD global segmentation quality (SegQ) score was 3.49 ± 0.51 out of 4, and the mean IQ score was 4.2 ± 0.7 out of 5, yielding a composite qualitative score (SegQ + 5-item checklist, range 1–9) of 7.3 ± 0.9 (Table 4). Clinically acceptable segmentations—defined as global SegQ ≥ 3 and checklist ≥ 3/5—were observed in 100% of cases (9/9). Representative examples of high- and low-performance 3D segmentations are shown in Fig. 6. In 3D VIBE MRI, correlation analysis showed a moderate-to-strong positive trend between image quality and segmentation performance (*r* = 0.64), although the association did not reach statistical significance (*p* = 0.064).

### Qualitative radiologist evaluation (pathology feasibility cohort)

The pathology feasibility cohort was assessed using the same qualitative scoring framework on 2D cine (20 images) and 3D static (10 volumes) and results are reported separately for 2D and 3D. Segmentations were rated clinically usable (SegQ ≥ 3/4) in 2D for 75% (15/20) and 70% (14/20) of the images (agreement 17/20) and in 3D in 80% (8/10) of the volumes for both readers (agreement 8/10) (Supplemental Fig. S9).

### Manual labeling effort and inference time

Manual annotation required 10 min per 2D image and 4.5 h per 3D volume (mean time). Mean automated inference time was 5 s per 2D image and under 30 s per 3D volume on a single graphics processing unit.

## Discussion

This study aimed to validate automatic diaphragm segmentation on thoracic MRI using complementary 2D cine bSSFP and 3D VIBE T1W-GRE sequences, with quantitative benchmarking and independent multireader qualitative review in adults. The choice of 2D cine bSSFP and 3D VIBE T1W-GRE reflects routine thoracic MRI protocols [10]: 2D bSSFP provides high temporal resolution for diaphragmatic motion assessment, whereas 3D T1W-GRE offers volumetric coverage to delineate thoracoabdominal interfaces [10, 11]. Together, they support functional and structural evaluation relevant to neuromuscular and respiratory disease and to thoracic oncology planning, while 4D MRI remains investigational and is not yet routinely implemented. Automating diaphragm delineation on these sequences is a key step toward scalable, standardized quantification that can be integrated into existing thoracic imaging workflows.

Reference masks were derived from exhaustive manual annotations by an experienced radiologist; given the workload, such labeling is impractical clinically. Automated inference was > 100× faster (5 s *versus* 10 min per 2D frame; 30 s *versus* 4.5 h per 3D volume). Because the diaphragm is a thin, widely extended musculotendinous surface that occupies only a small fraction of the total image volume, traditional volumetric overlap metrics—such as Dice similarity coefficient, accuracy, specificity—provide limited insight into segmentation quality.

Segmentation performance was therefore assessed using surface- and boundary-sensitive metrics (NSD, ASSD, HD95) [12–14]. Being less affected by class imbalance, these metrics better reflect boundary accuracy while remaining sensitive to local contour irregularities. ASSD and HD95 directly quantify contour alignment in mm, providing clinically meaningful measures of boundary accuracy, whereas NSD can be computed at predefined tolerances to account for small, clinically irrelevant deviations. To make NSD easier to interpret clinically, we reported it at a small, visually meaningful boundary tolerance. We used a 3-voxel tolerance (≈ 4.2 mm in 3D and ≈ 4.8 mm in 2D), which corresponds to a mild but clearly visible contour offset on diaphragm overlays. For context, diaphragm excursion during breathing is typically on the order of several cm, so a 3–5 mm boundary deviation represents a small fraction of expected displacement while remaining relevant for downstream motion biomarkers. Quantitative results were complemented by structured radiologist reviews to capture clinically important failure modes (discontinuities, leakage, temporal inconsistency). Finally, an RI-QUAL–derived IQ score linked artifact burden to segmentation confidence, supporting translation from technical performance to clinical usability [9, 15].

Both models achieved accurate boundary delineation (NSD@3 voxels, mean ± SD: 0.945 ± 0.076 for 2D and 0.922 ± 0.057 for 3D; ASSD: 1.97 ± 2.29 mm and 1.88 ± 1.97 mm; HD95: 9.89 ± 15.2 mm and 7.67 ± 6.3 mm, respectively). Performance may be further improved with postprocessing, particularly in motion-prone or low-contrast regions, yet it remains within or above ranges reported in prior indirect thoracic segmentation studies. Five-fold cross-validation showed consistent generalizability with low inter-fold variability. In addition, a small external feasibility cohort of patients with diaphragmatic dysfunction (*n* = 5) provided preliminary evidence of feasibility in pathology.

Historically, MRI assessments of diaphragmatic motion relied on indirect measures such as thoracic volume changes, paradoxical excursions, or disease-specific kinetics [4, 16–18]. More recently, lung boundary segmentation has been used to infer diaphragmatic motion indirectly, often achieving high Dice scores without delineating the muscle itself [19–21]. Only a few studies attempted direct segmentation, all in pediatric cohorts. Akhtar et al [7] reported HD95 = 3 ± 3 mm, Hao et al [22] focused on biomechanical indices, and Hosseini et al [23] reconstructed diaphragm surfaces indirectly from lung boundaries. To our knowledge, this provides one of the first adult references of direct diaphragm segmentation, allowing us to establish quantitative benchmarks for dynamic MRI. Boundary-based metrics (NSD, ASSD, HD95) offered a clinically interpretable assessment of contour accuracy. Although quantitative metrics capture geometric accuracy, they do not fully reflect clinical usability. In the complementary multireader review, 100% of 3D and 87.5% of 2D segmentations were rated clinically acceptable or requiring only minor adjustments. From a workflow perspective, this suggests a decision-support role, providing an initial contour that can be accepted or minimally edited rather than requiring full manual delineation. Most discrepancies clustered in the costophrenic recesses, where reduced image quality limited contour conspicuity, suggesting limited visibility rather than systematic algorithmic bias. Occasional false positives occurred in upper slices, with shoulder structures mislabelled as diaphragm due to similar intensity and curvature. These errors could be mitigated by rule-based postprocessing to remove non-diaphragmatic shoulder regions or, in future work, by enforcing anatomical continuity constraints to preserve connected diaphragmatic surfaces. Moderate-to-strong correlations between image quality and segmentation performance (r = 0.64 in 3D; r = 0.65 in 2D) indicate that higher image quality is associated with improved segmentation accuracy, but they also suggest that image quality alone does not fully explain segmentation performance. These findings underscore the importance of acquisition quality for reliable automated diaphragm analysis, particularly given the practical difficulty of obtaining optimal images in pathological or less cooperative patients. Overall, concordance between boundary-based metrics and expert assessment supports the feasibility of anatomically consistent diaphragm segmentations and justifies further multicenter evaluation toward workflow integration.

Across modalities, diaphragm analysis has reached mm-level performance, yet direct delineation remains challenging. CT studies report 2–4 mm errors [24, 25], and deep-learning pipelines such as Diaphragm-Net achieve a Dice similarity coefficient > 0.90 [26, 27]. In cone-beam computed tomography, radiotherapy-oriented diaphragm tracking reaches < 5-mm accuracy [28], while ultrasound models automate excursion or thickening-fraction estimation with Dice similarity coefficient ≈ 0.85 and < 10% error [29]. In this context, our MRI-based approach enables direct muscle-level segmentation and demonstrates technical feasibility and preliminary clinical acceptability in a small pathology cohort (*n* = 5) with > 70% of segmentations rated clinically usable. Automated segmentation is a prerequisite for reproducible diaphragm-motion biomarkers; notably, MRI-derived respiratory length–area ratio metrics can detect subclinical diaphragmatic weakness in Pompe disease and track therapy response [6], supporting multimodal structure–function assessment when combined with pulmonary function testing [30].

Strengths include the study design, two U-Net models tailored to routine 2D cine and 3D MRI, combined quantitative and radiologist-based evaluation, and clinically acceptable inference times (< 30 s/volume). The 3D model was stable and relatively insensitive to image noise. Limitations include the single-center setting, predominantly healthy cohort, a single-radiologist reference standard, and internal testing, which may restrict generalizability—particularly to patients with complex disease and lower-quality imaging. Excluding nondiagnostic acquisitions may have introduced selection bias toward diagnostically usable images. Primary model development and validation were performed in subjects without known or suspected diaphragmatic dysfunction to establish a controlled baseline; therefore, segmentation performance in patients with diaphragmatic abnormalities remains to be verified. The overall study should be considered a proof-of-concept for automated diaphragm segmentation in a controlled population. The small pathology cohort (*n* = 5) was included for feasibility assessment only and does not constitute clinical validation. Future work will expand multicenter, disease-cohort validation and assess robustness across sequences, vendors field strengths, and routine clinical acquisition variability. Although the 2D and 3D models share a common design, the 2D approach processed cine frames independently and underperformed the 3D model, motivating spatiotemporal (2D+t) modeling to exploit inter-frame continuity.

Although large-scale segmentation frameworks such as TotalSegmentator and foundation models [31–36] achieve impressive multi-organ performance, none currently delineate the diaphragm, highlighting the need for dedicated solutions [37]. By providing dynamic MRI benchmarks, our study helps fill this gap and establishes the basis for spatiotemporal biomarkers with potential clinical impact. Importantly, this study evaluates segmentation performance and does not yet report functional endpoints (cranio-caudal excursion, dome displacement, or thoracoabdominal volume change) across respiratory phases. Nevertheless, robust automated delineation is a key enabler of scalable, standardized diaphragm-motion quantification, with potential to reduce manual workload and support objective assessment and longitudinal monitoring. Subsequent work will derive functional measures from the automated masks and extend validation to multicenter and disease cohorts, with Picture Archiving and Communication System/Radiology Information System integration and potential gains in data efficiency through spatiotemporal transfer learning [38, 39].

In conclusion, this study establishes a framework for direct diaphragm segmentation in adults on routine MRI, spanning dynamic 2D cine and static 3D sequences with multireader evaluation and boundary-oriented metrics. The proposed approach delivers fast and standardized diaphragm segmentation from routine MRI. Further validation in larger pathological and multicenter cohorts is needed to assess robustness and potential clinical applicability.

## Supplementary information

**Additional file 1: Supplemental Figure 1:** Study flow diagram: patient inclusion and imaging dataset. **Supplemental Table 1:** MRI acquisition parameters. **Supplemental Figure 2:** Manual segmentation of left and right diaphragm from a 2D acquisition. **Supplemental Figure 3:** Manual segmentation of the diaphragm from a 3D-UTE acquisition. **Supplemental Figure 4:** Manual segmentation of the diaphragm from a 3D VIBE T1w-GRE acquisition. **Supplemental Table 2:** Annotated datasets splitting for U-Net training, validation and testing. **Supplemental Figure 5:** Illustration of the segmentation 2D dataset creation. **Supplemental Figure 6:** 2D U-Net model diagram. **Supplemental Figure 7:** Illustration of the segmentation 3D-UTE dataset creation. **Supplemental Figure 8:** Illustration of the segmentation 3D dataset creation. **Supplemental Table 3:** Summary of architecture and training parameters for the 2D, 3D U-Net models. **Supplemental Table 4:** IQ Scoring Card adapted from the RI-QUAL framework [9]. **Supplemental Video 1:** 3D visualization of automated diaphragm segmentation. The lung–abdominal boundary surfaces (blue) were extracted from thoracic MRI using the 3D-UTE U-Net model across 32 respiratory phases. **Supplemental Video 2:** 2D dynamic visualization of automated diaphragm segmentation. The right and left diaphragmatic surfaces (blue) were extracted from thoracic MRI using the 2D U-Net model in sagittal views during dynamic forced respiration. **Supplemental Figure 9:** Fully automated diaphragm segmentation in patients with diaphragmatic dysfunction. (a-b) Patient with left hemidiaphragm dysfunction and dome elevation: (a) 3D T1w-GRE volume and (b) the corresponding 2D cine bSSFP slice of the same hemidiaphragm; (c-d) Patient with right hemidiaphragm dysfunction and dome elevation: (c) 3D T1w-GRE volume and (d) the corresponding 2D cine bSSFP slice of the same hemidiaphragm. It illustrates consistent delineation across 3D and 2D acquisitions.

## Data Availability

The datasets generated and/or analyzed during the current study are not publicly available due to institutional and privacy restrictions but are available from the corresponding author on reasonable request.

## Abbreviations

2D: Two-dimensional
2D+t: Two-dimensional plus time (spatiotemporal)
3D: Three-dimensional
4D: Four-dimensional
ASSD: Average symmetric surface distance
bSSFP: Balanced steady-state free precession
CT: Computed tomography
HD95: 95th percentile Hausdorff distance
IQ: Image quality
MRI: Magnetic resonance imaging
NSD: Normalized surface Dice
RI-QUAL: Radiological image quality score
SD: Standard deviation
SegQ: Segmentation quality (score)
T1W-GRE: T1-weighted gradient-echo
U-Net: U-Net (convolutional neural network architecture)
VIBE: Volumetric interpolated breath-hold examination
